# Inhibitory Effects of Gyeongok-go on Lung Injury in a Chronic Obstructive Pulmonary Disease Mouse Model

**DOI:** 10.3390/ph19040618

**Published:** 2026-04-14

**Authors:** Won-Kyung Yang, Jin Kwan Choi, Seung-Hyung Kim, Su Won Lee, Yee Ran Lyu, Yang-Chun Park

**Affiliations:** 1 Institute of Traditional Medicine and Bioscience, Daejeon University, Daejeon 34520, Republic of Korea; ywks1220@dju.kr (W.-K.Y.); sksh518@dju.kr (S.-H.K.); 2 Division of Respiratory Medicine, Department of Internal Medicine, College of Korean Medicine, Daejeon University, Daejeon 34520, Republic of Korea; poporang1218@naver.com (J.K.C.); tndnjs3325@daum.net (S.W.L.); 3 KM Science Research Division, Korea Institute of Oriental Medicine, Daejeon 34054, Republic of Korea; onedoctor2ran@kiom.re.kr

**Keywords:** Gyeongok-go, chronic obstructive pulmonary disease, cigarette smoke extract, lipopolysaccharide, lung injury

## Abstract

**Background/Objectives:** Chronic obstructive pulmonary disease (COPD) is characterized by incomplete recovery of airflow blockage; however, effective therapeutic agents that can prevent lung function deterioration are limited. East Asian herbal treatments have gained attention for their potential benefits in managing COPD. This study aimed to evaluate the inhibitory effects of Gyeongok-go (GOG) on lung injury in a COPD mouse model. **Methods:** Lipopolysaccharide (LPS)-induced alveolar macrophage (MH-S) cells were treated with GOG (50, 100, 200, and 400 μg/mL), and analyzed using enzyme-linked immunosorbent assay (ELISA). C57BL/6 mice were challenged with cigarette smoke extract and LPS and then treated with vehicle only, dexamethasone (3 mg/kg), or GOG (100, 200, or 400 mg/kg). Bronchoalveolar lavage fluid (BALF) or lung tissues were analyzed using cytospin, ELISA, real-time PCR, flow cytometry, hematoxylin and eosin, and Masson’s trichrome staining. **Results:** Treatment with GOG decreased tumor necrosis factor-alpha (TNF-α) and interleukin (IL)-6 expression in LPS-challenged MH-S cells. In COPD mice, GOG significantly decreased the elevated numbers of neutrophils, total cells, macrophages, and Gr-1^+^/Siglec-F, Gr-1^+^/CD11b^+^, and CD44^high^/CD62L^−^ cells. It also downregulated the expression of TNF-α, IL-17A, macrophage inflammatory protein-2 (MIP2), and CXC chemokine ligand-1 in BALF. GOG also inhibited the increase in *Mip2*, *Cox-2*, and *Trpv1* mRNA expression. Moreover, GOG prevented the increase in the number of total cells, neutrophils, Gr-1^+^/Siglec-F, Gr-1^+^/CD11b^+^, CD44^high^/CD62L^−^, and CD21^+^/CD35^+^/B220^+^ cells in lung tissues. Notably, GOG decreased the severity of lung injury. **Conclusions:** Overall, these findings indicate that GOG alleviates lung injury, suggesting its potential in the treatment of COPD.

## 1. Introduction

Chronic obstructive pulmonary disease (COPD), a lung disease characterized by incomplete recovery of airflow blockage, can be caused by various factors, including smoking, occupational exposure, infection, and air pollution, leading to abnormalities in the airway and parenchyma [1]. The prevalence and mortality of COPD are continually rising, owing to constant exposure to risk factors and aging. According to the Global Burden of Disease Study, COPD resulted in the death of 3.2 million people in 2017, making it the seventh major cause of death, accounting for 50% of the 545 million patients with chronic respiratory disease [2]. COPD is characterized by irreversible airflow obstruction involving chronic inflammation of the small airways. This obstruction leads to airway stenosis caused by structural changes, such as fibrosis, narrowing, or blockage of the airway. These changes occur during expiratory blockage due to reduced lung elasticity of the lungs resulting from parenchymal destruction [3]. While COPD is characterized by pathological changes primarily affecting the distal small airway and parenchyma, it can also involve the proximal central airways and pulmonary arteries. It manifests in the forms of small airway disease, chronic bronchitis, and emphysema, and is characterized by airway stenosis resulting from chronic inflammation, tissue damage, and parenchymal destruction [4]. Specifically, chronic inflammation plays a central role in the onset and progression of COPD. A persistent inflammatory response triggers pathological changes in COPD through various mechanisms, such as oxidative stress and protease/antiprotease imbalance [4,5]. According to the GOLD 2026 report, Chronic Obstructive Pulmonary Disease (COPD) remains a leading cause of morbidity and mortality worldwide, now recognized as the third leading cause of death globally. The report highlights that the global burden of COPD is increasing due to the continued prevalence of smoking and rising exposure to environmental pollutants and biomass fuels in aging populations. Notably, the GOLD 2026 update emphasizes ‘pre-COPD’ and ‘PRISm’ as critical early stages for intervention, reinforcing the need for preventive therapeutic strategies like Gyeongok-go to mitigate lung inflammation before irreversible structural damage occurs. Moreover, chronic exposure of the respiratory system to harmful substances, such as cigarette smoke, causes an inflammatory response, leading to an increased number of active neutrophils, macrophages, and CD8^+^ T lymphocytes across various regions of the lung [6,7]. For example, patients with COPD exhibit an elevated number of active neutrophils in the sputum or bronchoalveolar lavage fluid (BALF), which secrete proteases such as neutrophil elastase, neutrophil cathepsin G, and neutrophil proteinase-3. These enzymes contribute to the development of emphysema by degrading major components of parenchymal connective tissues, such as elastin [8]. Furthermore, the number of macrophages is significantly increased in the airway, parenchyma, BALF, and sputum of patients with COPD. These macrophages promote an inflammatory response by secreting inflammatory mediators, such as tumor necrosis factor-alpha (TNF-α), chemokine ligand-1 (CXCL-1), CXCL-8, CCL2, and LTB_4_, promoting the onset of COPD [9]. CD8^+^ T lymphocytes further sustain the inflammatory state by secreting proteases, such as perforin, granulysin, and granzyme-A/B, and inducing cytolysis and apoptosis in the alveolar epithelial cells and vascular endothelial cells [10]. Therefore, an inflammatory response activates macrophage phagocytosis, induces neutrophils by producing macrophage inflammatory protein-2 (MIP-2) and CXC, and destroys lung tissues by inducing the secretion of proteases such as matrix metalloproteinases [11]. Furthermore, TNF-α is a major inflammatory cytokine that increases the inflammatory response in the airway and parenchyma. IL-6 further exacerbates the inflammatory response of COPD by inhibiting the signaling of regulatory T cells [12]. Overall, this highlights the role of inflammation in COPD.

Current treatment strategies rely on a bronchodilator, a beta2 agonist, which improves lung function by relaxing airway smooth muscles [13]. Moreover, combined inhalational therapy can decrease the rate of decline of lung function, with recent research emphasizing the importance of maximizing bronchodilation in COPD with inhaled dual-bronchodilator treatment, which may further enhance patient-related outcomes [14]. However, this approach may also increase local adverse effects, such as oropharyngeal mycosis, voice changes, and intermittent coughing, which are caused by upper airway stimulation, as well as systemic side effects, including adrenal suppression, bruises, decreased bone density, and pneumonia [15]. Therefore, an effective and safe therapeutic agent that can prevent lung function deterioration in the long term while regulating symptoms without side effects is urgently needed [11].

East Asian herbal treatments have gained attention for their potential benefits in managing COPD. Previous studies have examined the effects of formulations such as Socheongryong-tang [16,17], Saengmaekcheongpye-eum [18], Maekmoondong-tang [18], Cheongsangboha-tang-derived PM014 [19], Xiegan-tang [20], Xiegan-tang-derived SGX01 [21], Gwaruhaengryeon-hwan-derived GHX02 [22], and Gamgil-tang-derived GGX [23]. Among these, Gyeongok-go (GOG) comprises four herbs: Saengjihwang, Insam, Bokryeong, and Bongmil. Notably, GOG is perceived as a universal nutritional tonic in Bangyakhappyeon [24], and its anti-fatigue [18,25], growth-promoting [26,27,28], anti-oxidant [29,30,31,32], and anti-inflammatory [16,33] therapeutic effects against climacteric syndrome [34] and skin disorders [35,36] have been investigated. However, the effects of GOG on respiratory diseases and tubercle bacilli [37], as well as its anti-inflammatory and antitussive expectorant effects [38], remain unclear.

In the present study, we aimed to explore the effects of GOG on respiratory diseases by assessing its efficacy in a COPD mouse model. First, we performed in vitro experiments to determine whether GOG could inhibit lipopolysaccharide (LPS)-induced cytokine expression in MH-S cells derived from mouse alveolar macrophages. In a cigarette smoke extract (CSE)- and LPS-induced COPD animal model, we assessed the effect of GOG on neutrophil differentiation using cytosine and analyzed the expression levels of related cytokines via enzyme-linked immunosorbent assay (ELISA). We also investigated the effects of GOG on related immune cells using fluorescence-activated cell sorting. Moreover, we quantified the expression of related genes via real-time polymerase chain reaction (RT-PCR). Finally, we verified suppression of lung tissue damage by histologically analyzing mouse lung tissue.

## 2. Results

### 2.1. Tentative Identification of Major Compounds in GOG via Ultra-Performance Liquid Chromatography (UPLC) with Quadrupole Time-of-Flight Mass Spectrometry (QTOF-MS) Analysis

**Figure 1:**
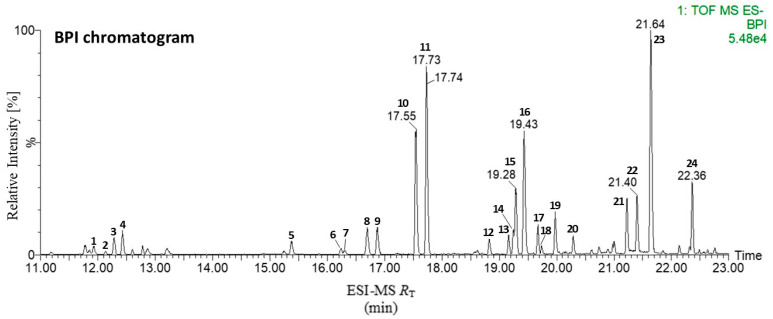
Representative base peak intensity chromatogram of the Gyeongok-go extract in the negative ion mode. The peak (1–24) information is listed in Table S1.

We tentatively identified 24 major compounds as known chemical constituents via HR-ESI-QT of MS analysis (Figure 1). Peaks **1**–**11**, **15**, and **16** corresponded to ginseng saponins (commonly known as ginsenosides) belonging to the triterpene saponin class of representative natural steroid glycosides in *Panax ginseng*. In the negative ion mode, we detected 13 ginsenosides as [M − H]^−^ ions and [M + COOH]^−^ ions with high mass accuracy (<3.3 ppm). We identified 13 peaks as ginsenoside 20(*S*)-Rh_1_ (**1**), Rg_2_ (**2**), 20(*R*)-Rh_1_ (**3**), Ro (**4**), F_2_ (5), F_4_ (**6**), Rk_3_ (**7**), Rh_4_ (**8**), R_1_ (**9**), 20(*S*)-Rg_3_ (**10**), 20(*R*)-Rg_3_ (**11**), Rk_1_ (**15**), and Rg_5_ (**16**). Peaks **12**–**14** and **17**–**24** corresponded to triterpene acids from *Poria cocos* in the negative ion mode. We unequivocally identified 11 peaks, namely 16α-hydroxydehydrotrametenolic acid (**12**), poricoic acid B (**13**), dehydrotumulosic acid (**14**), poricoic acid A (**17**), poricoic acid G (**18**), polyporenic acid C (**19**), 16α-hydroxyeburiconic acid (**20**), 3-*O*-acetyl-16α-hydroxytrametenolic acid (**21**), dehydropachymic acid (**22**), pachymic acid (**23**), and dehydrotrametenolic acid (**24**), by comparing high mass accuracy (<1.4 ppm). We also tentatively identified 24 major compounds (**1**–**24**) by analyzing spectroscopic data, including HR-MS (accurate mass in negative mode), HR-MS/MS spectra (fragmentation pattern), and UV/Vis spectra (absorption maxima), and comparing them with the published literature [39,40,41]. Information on the peak number, retention time (*R*_T_), detected ion (adduct), mass accuracy (ppm error), molecular formula, fragment, and compound name has been deposited in the library for the 24 compounds identified in GOG. The Gyeongok-go (GOG) extract was standardized by monitoring its primary marker compounds, ensuring they met the quality control standards of the Korean Pharmacopoeia. This standardization ensures that the observed pharmacological effects are consistent with the known chemical profile of GOG.

### 2.2. Effects of GOG In Vivo and In Vitro

**Figure 2:**
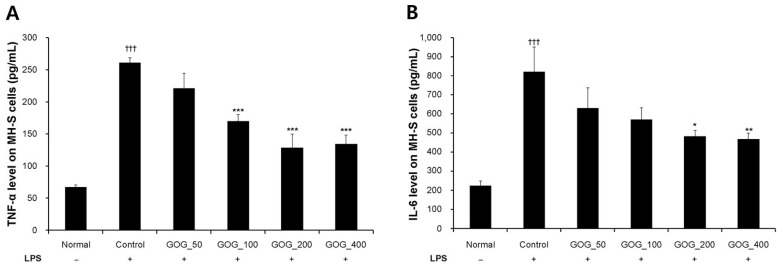
Effects of GOG on TNF-α (**A**) and IL-6 (**B**) production in LPS-induced MH-S cells. MH-S cells were challenged with LPS (Control, 400 ng/mL) and then treated with GOG at 50, 100, 200, and 400 µg/mL. Dexamethasone (10 µM) was used as a positive control. Data are presented as the mean ± standard deviation. Significantly different from the normal group (††† *p* < 0.001); Significantly different from control group (* *p* < 0.05, ** *p* < 0.01, *** *p* < 0.001). GOG, Gyeongok-go; TNF, tumor necrosis factor; IL-6, interleukin 6; LPS, lipopolysaccharide.

**Figure 3:**
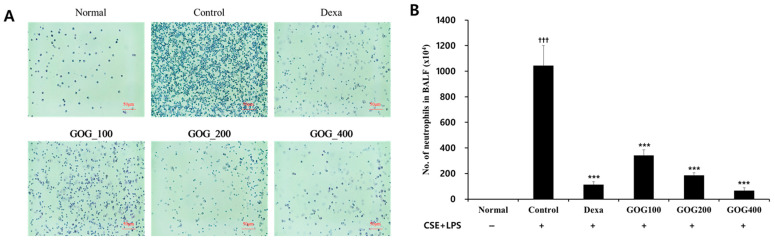
Effect of GOG on cytospin (**A**) and the number of neutrophils (**B**) in the BALF of mice with COPD. Mice were induced via CSE+LPS aspiration (Control) and then treated with dexamethasone 3 mg/kg (Dexa) and GOG (100, 200, and 400 mg/kg) for 21 days. Data are presented as the mean ± standard error (SE) (*n* = 8). Significant difference compared with the normal group (††† *p* < 0.001); Significant difference compared with the control group (*** *p* < 0.001). GOG, Gyeongok-go; BALF, bronchoalveolar lavage fluid; COPD, chronic obstructive pulmonary disease; CSE+LPS, intranasal instillation of cigarette smoke extract (CSE, 1 mg/mL) and lipopolysaccharide (LPS, 100 µg/mL).

**Figure 4:**
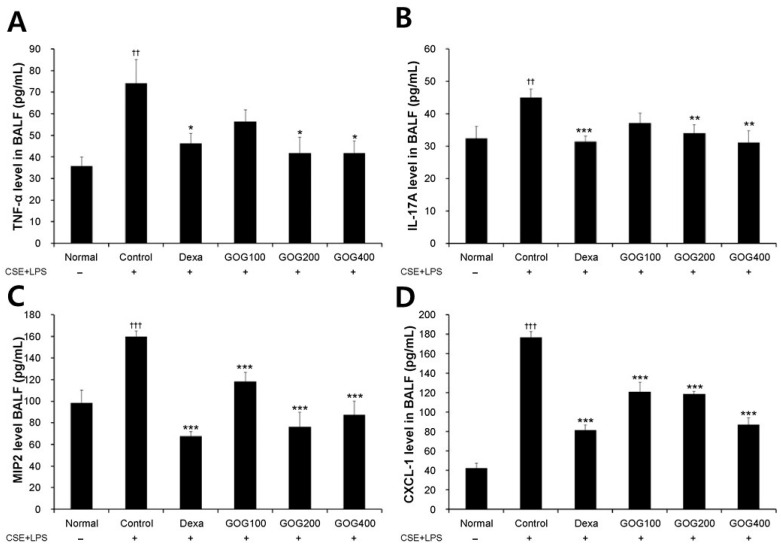
Effect of GOG on TNF-α (**A**), IL-17 (**B**), MIP2 (**C**), and CXCL- (**D**) production in the BALF of mice with COPD. Mice were induced via CSE+LPS aspiration (Control) and treated with dexamethasone 3 mg/kg (Dexa) and GOG (100, 200, and 400 mg/kg) for 21 days. Expression of TNF-α was determined using ELISA. Data are presented as the mean ± SE (*n* = 8). Significant difference compared with the normal group (†† *p* < 0.01, ††† *p* < 0.001); Significant difference compared with the control group (* *p* < 0.05, ** *p* < 0.01, *** *p* < 0.001). GOG, Gyeongok-go; TNF-α, tumor necrosis factor alpha; IL-17, interleukin 17; MIP2, mouse macrophage inflammatory protein 2; CXCL-1, C-X-C motif chemokine ligand 1; BALF, bronchoalveolar lavage fluid; COPD, chronic obstructive pulmonary disease; CSE+LPS, intranasal instillation of cigarette smoke extract (CSE, 1 mg/mL) and lipopolysaccharide (LPS, 100 µg/mL).

**Figure 5:**
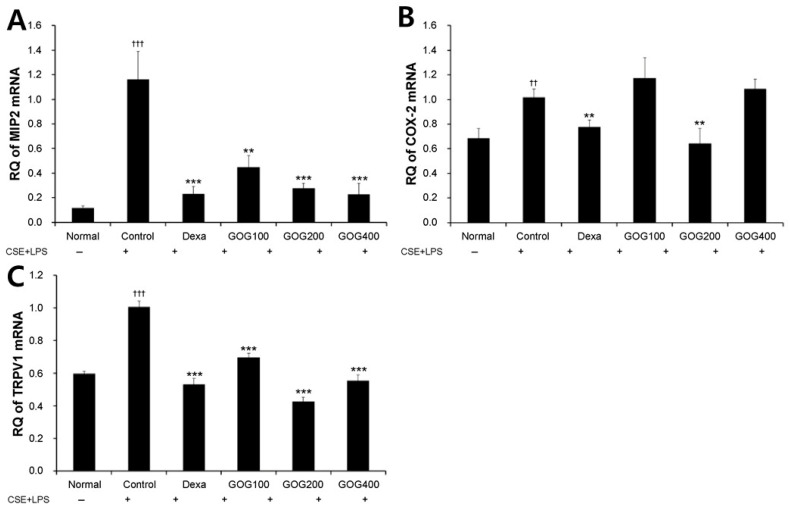
Effect of GOG on Mip2 (**A**), Cox-2 (**B**), and Trpv1 (**C**) mRNA expression in the lung tissue of mice with COPD. COPD was induced via CSE+LPS aspiration (Control) and treated with dexamethasone 3 mg/kg (Dexa) and GOG (100, 200, and 400 mg/kg) for 21 days. Expression of Mip2 was determined using real-time polymerase chain reaction. Data are presented as the mean ± SE (*n* = 8). Significant difference compared with the normal group (†† *p* < 0.01, ††† *p* < 0.001); Significant difference compared with control group (** *p* < 0.01, *** *p* < 0.001). GOG, Gyeongok-go; MIP2, mouse macrophage inflammatory protein 2; COX-2, cyclooxygenase-2; TRPV1, transient receptor potential vanilloid 1; COPD, chronic obstructive pulmonary disease; CSE+LPS, intranasal instillation of cigarette smoke extract (CSE, 1 mg/mL) and lipopolysaccharide (LPS, 100 µg/mL).

**Figure 6:**
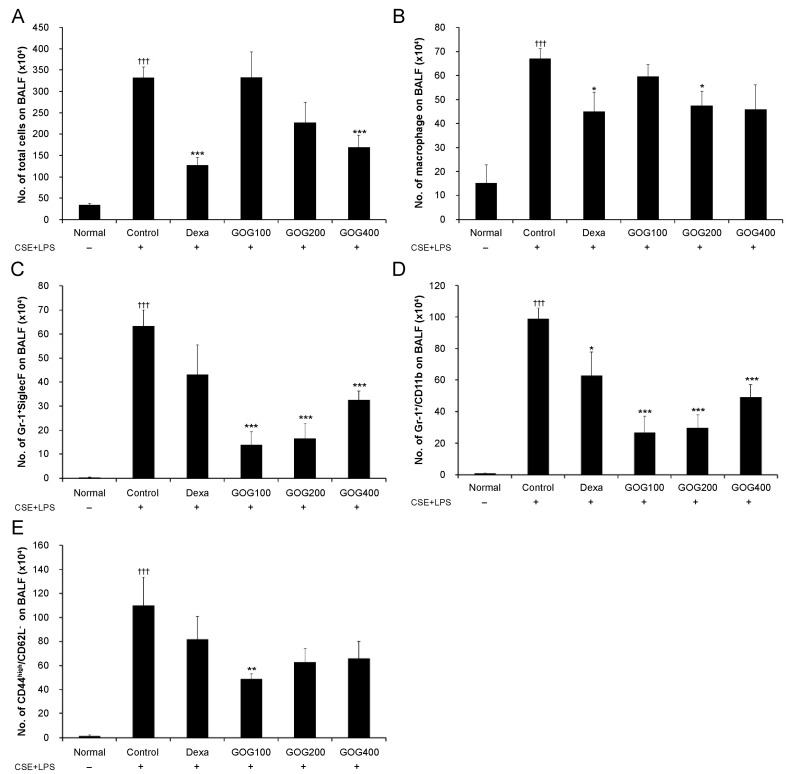
Effect of GOG on various immune cell subtypes in BALF of mice with COPD. The absolute numbers of total cells (**A**), macrophages (**B**), Gr-1+/Siglec-F (**C**), Gr-1+/CD11b+ (**D**), and CD44high/CD62L- (**E**) cells were determined via flow cytometry. Mice were induced via CSE+LPS aspiration (Control) and treated with dexamethasone 3 mg/kg (Dexa) and GOG (100, 200, and 400 mg/kg) for 21 days. Data are presented as the mean ± SE (*n* = 8). Significant difference compared with the normal group (††† *p* < 0.001); Significant difference compared with control group (* *p* < 0.05, ** *p* < 0.01, *** *p* < 0.001). GOG, Gyeongok-go; BALF, bronchoalveolar lavage fluid; COPD, chronic obstructive pulmonary disease; CSE+LPS, intranasal instillation of cigarette smoke extract (CSE, 1 mg/mL) and lipopolysaccharide (LPS, 100 µg/mL).

To evaluate the fundamental anti-inflammatory potential of GOG in alveolar macrophages, we used LPS-induced MH-S cells as a standardized screening model. The expression of TNF-α and interleukin-6 (IL-6) was significantly increased in the LPS-challenged control group compared to the normal group. In contrast, GOG treatment (50, 100, 200, or 400 µg/mL) significantly and dose-dependently inhibited the production of TNF-α (Figure 2A) and IL-6 (Figure 2B) compared to the control group. In this study, the initial in vitro assays (Figure 2) were designed as a preliminary dose–response screening to evaluate the broad anti-inflammatory potential of GOG. While a specific pharmacological positive control was not included in this baseline screening phase, the robust and statistically significant induction of TNF-α and IL-6 by LPS confirmed the responsiveness of the MH-S cell system. Furthermore, the experimental validity of our disease model and the potency of GOG were systematically validated in subsequent assays (Figure 3, Figure 4, Figure 5 and Figure 6), where dexamethasone was employed as a positive control and exhibited the expected inhibitory effects, thereby providing an internal benchmark for the efficacy of GOG.

The proportion of neutrophils was significantly increased in the COPD-induced control MH-S cells compared to that in the normal group. However, it significantly decreased in the positive control and experimental groups treated with 100, 200, or 400 µg/mL GOG compared to that in the control group (Figure 3). Moreover, TNF-α and IL-17 expression levels were significantly higher in the COPD-induced control MH-S cells than in the normal group (*p* < 0.01). However, they were significantly decreased in the experimental groups treated with 200 and 400 µg/mL GOG compared to the control group (TNF-α, *p* < 0.05, Figure 4A; IL-17, *p* < 0.01, Figure 4B). MIP2 (Figure 4C) and mouse chemokine ligand-1 (CXCL-1; Figure 4D) expression levels were significantly higher in the COPD-induced control group than in the normal group (*p* < 0.001). In contrast, they were significantly lower in the positive control and experimental groups treated with 100, 200, and 400 µg/mL GOG than in the control group (*p* < 0.001).

The relative mRNA expression levels of *Mip2* (Figure 5A) and *Trpv1* (Figure 5C) were significantly increased in the COPD-induced control group and significantly decreased in the positive control group (*p* < 0.001) compared to those in the normal group (*p* < 0.001). Notably, *Mip2* and *Trpv1* expression levels exhibited a significant significantly inhibited decrease in the experimental groups treated with 100, 200, and 400 µg/mL GOG (*p* < 0.001) compared to those in the control group (Figure 5).

The total number of cells in the BALF was significantly higher in the control group than in the normal group and significantly lower in the positive control and experimental group treated with 400 µg/mL GOG. In terms of immune cell subtypes, compared to those observed in the control group, the number of macrophages was significantly decreased in the experimental group treated with 400 µg/mL GOG, the numbers of Gr-1^+^/sialic acid-binding immunoglobulin-like lectin F (Siglec-F) and Gr-1^+^/CD11b^+^ cells were significantly decreased in all the experimental groups treated with GOG, and that of CD44^high^/CD62L^−^ cells was significantly decreased in the experimental group treated with 100 µg/mL GOG (Figure 6).

### 2.3. Effects of GOG on Immune Cell Activation in Lung Tissue

**Figure 7:**
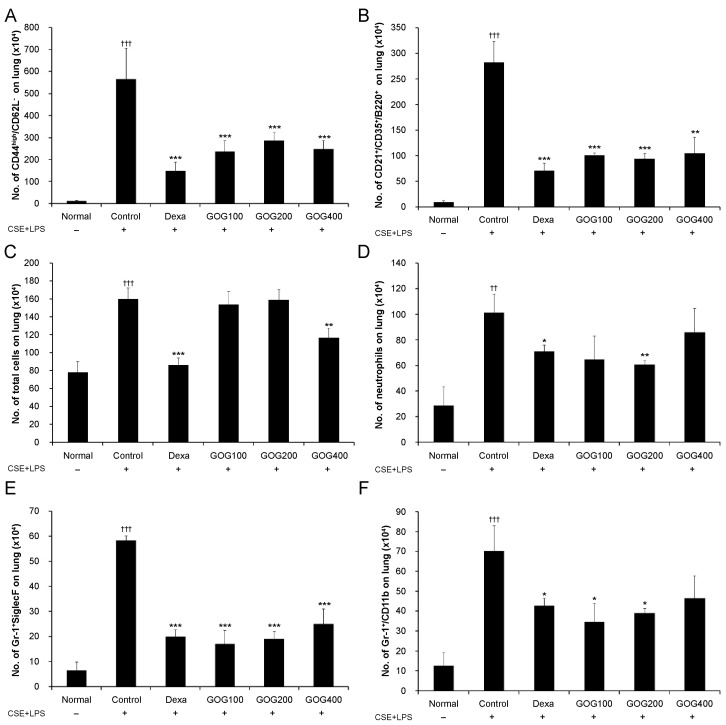
Effect of GOG on various immune cell subtypes in the lung tissue of mice with COPD. The absolute numbers of total cells (**A**), neutrophils (**B**), Gr-1+/Siglec-F (**C**), Gr-1+/CD11b+ (**D**), CD44high/CD62L- (**E**), and CD21+/CD35+/B220+ (**F**) cells were determined via flow cytometry. Mice were induced via CSE+LPS aspiration (Control) and treated with dexamethasone 3 mg/kg (Dexa) and GOG (100, 200, and 400 mg/kg) for 21 days. Data are presented as the mean ± SE (*n* = 8). Significant difference compared with the normal group (†† *p* < 0.01, ††† *p* < 0.001); Significant difference compared with the control group (* *p* < 0.05, ** *p* < 0.01, *** *p* < 0.001). GOG, Gyeongok-go; COPD, chronic obstructive pulmonary disease; CSE+LPS, intranasal instillation of cigarette smoke extract (CSE, 1 mg/mL) and lipopolysaccharide (LPS, 100 µg/mL).

In lung tissue, the total number of cells was significantly higher in the control group but lower in the positive control and experimental groups treated with 100, 200, and 400 µg/mL GOG than in the normal group. The decrease observed in the 400 µg/mL treatment group was statistically significant. The immune cell subtype analysis revealed the number of neutrophils was significantly decreased in the experimental group treated with 200 µg/mL GOG, that of Gr-1^+^/CD11b^+^ cells (Figure 7F) was significantly decreased in the experimental groups treated with 100 and 200 µg/mL GOG, and that of Gr-1^+^/Siglec-F (Figure 7E) and CD44^high^/CD62L^−^ cells (Figure 7A) was significantly decreased in all experimental groups treated with GOG compared to those in the control group (Figure 7).

### 2.4. Effects of GOG on Lung Tissue Histology

**Figure 8:**
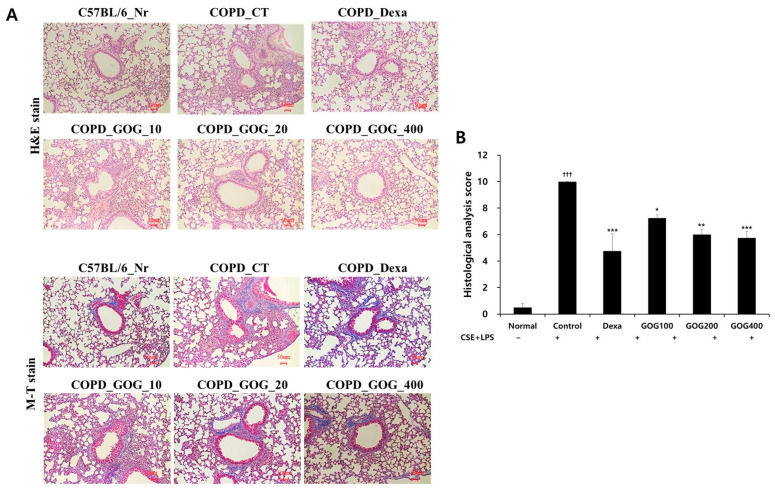
Effect of GOG on histopathological changes in the lungs of mice with COPD. Mice were induced via CSE+LPS aspiration (Control) and treated with dexamethasone 3 mg/kg (Dexa) and GOG (100, 200, and 400 mg/kg) for 21 days. (**A**) Representative sections of lungs stained with hematoxylin and Eosin and Masson’s trichrome stain (light microscope at 100× magnification). (**B**) Quantitative evaluation of the degree of lung tissue damage. Data are presented as the mean ± SE (*n* = 8). Significant difference compared with the normal group (††† *p* < 0.001); Significant difference compared with the control group (* *p* < 0.05, ** *p* < 0.01, *** *p* < 0.001). GOG, Gyeongok-go; COPD, chronic obstructive pulmonary disease; CSE+LPS, intranasal instillation of cigarette smoke extract (CSE, 1 mg/mL) and lipopolysaccharide (LPS, 100 µg/mL).

After the completion of the in vivo experiments, we conducted H&E staining of the lung tissue of COPD mice, which revealed an inconsistent alveolar size. Moreover, collagen deposition, airway wall hyperplasia, and cell deposition around the alveolus were prominent in the control group compared to the normal group. The morphology of the alveoli was relatively consistent in the positive control group. In the experimental groups treated with GOG, the cell deposition around the alveoli was reduced, and alveolar morphology was relatively consistent compared to that in the control group. In addition, we performed Masson’s Trichrome staining to assess the level of alveolar cell damage through collagen deposition. In the control group, we observed infiltration of macrophages and blood cells and goblet cell proliferation in the bronchioles and alveoli. In the experimental groups treated with GOG, the proportion of active macrophages was decreased, and goblet cell proliferation and inflammatory cell infiltration were suppressed to levels approaching those observed in the normal group. Quantitative scoring of lung tissue damage revealed a significant increase in the control group and a significant decrease in the positive control group compared to the normal group. Notably, the damage in the experimental groups treated with 100, 200, and 400 µg/mL GOG was significantly alleviated (Figure 8).

**Figure 9:**
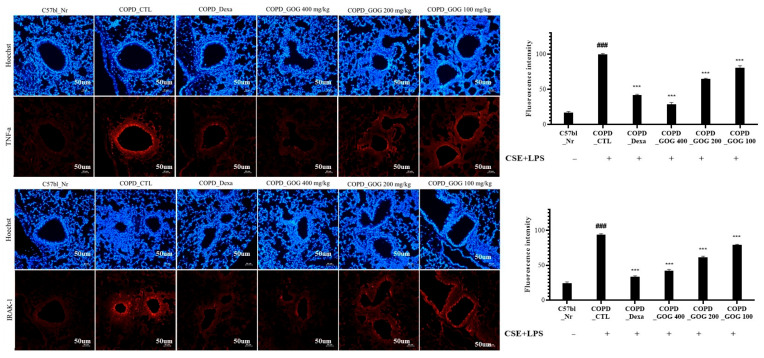
Effects of GOG on the histology of the lungs of mice with COPD. Mice were induced via CSE+LPS aspiration (Control) and treated with dexamethasone 3 mg/kg (Dexa) and GOG (100, 200, and 400 mg/kg) for 21 days. GOG, Gyeongok-go; COPD, chronic obstructive pulmonary disease; CSE+LPS, intranasal instillation of cigarette smoke extract (CSE, 1 mg/mL) and lipopolysaccharide (LPS, 100 µg/mL) (microscope at 100× magnification) (### *p* < 0.001, *** *p* < 0.001).

We also analyzed the expression levels of proteins involved in signal transduction in lung tissue using immunohistochemistry. The expression of TNF-α and IRAK-1 was significantly increased in the control group compared to that in the normal group. Notably, TNF-α and IRAK-1 expression exhibited a significantly inhibited decrease in the experimental groups treated with GOG compared to that in the control group (Figure 9).

## 3. Discussion

COPD has a high global prevalence and mortality, largely owing to its progressive nature and related diseases, leading to substantial socio-economic burden and a decline in quality of life [42,43]. GOG is widely used as a universal nutritional tonic that can also be used for treating chronic lung disease, given its indication for consumptive respiratory disease [44]. Studies on COPD have used animal models of lung injury induced by various methods, such as elastase [16,17], LPS [45,46], and inhalation of cigarette smoke [16]. In the present study, we investigated the effects of GOG on COPD using an animal model that can be uniformly challenged with smoking, the most critical virulence factor of COPD. Regarding the study design, while the in vivo model utilized a synergistic CSE+LPS challenge to mimic the complexity of COPD exacerbations, the in vitro experiments focused on LPS-induced MH-S cell activation. This approach was intended to first validate the direct anti-inflammatory efficacy of GOG against a potent, standardized macrophage stimulus. Although the in vitro model does not fully replicate the synergy of CSE and LPS, the consistent suppression of key pro-inflammatory cytokines (TNF-α and IL-6) across both models suggests that the therapeutic effects of GOG are robust and target the fundamental pathways of alveolar inflammation.

While we monitored cell morphology and relied on the established safety profile of GOG, the lack of a quantitative cell viability assay for the MH-S cell experiments is a limitation of this study. Although the consistent levels of internal controls (GAPDH) and total protein suggests no gross cytotoxicity, the possibility that high-dose GOG might affect cell metabolic activity cannot be entirely ruled out. Despite the significant findings, this study has several limitations. First, the 21-day CSE+LPS model may not fully represent the long-term, progressive nature of chronic airflow obstruction seen in human COPD patients. Second, while we observed the anti-inflammatory and lung-protective effects of GOG, the precise molecular targets and signaling pathways of its diverse bioactive components remain to be further elucidated. Lastly, although GOG has been used clinically for centuries, further well-designed clinical trials are necessary to confirm its efficacy and safety in COPD patients. Future studies focusing on long-term administration and detailed pharmacokinetic profiles will provide a more comprehensive understanding of GOG’s therapeutic potential. We adopted previously published methods [19,20], in which LPS and CSE are aspirated into the airway of C57BL/6 mice, to establish the mouse model. We first verified the inhibitory effects of GOG on LPS-induced TNF-α and IL-6 expression in MH-S cells in vitro. TNF-α and IL-6 are major inflammatory cytokines known to be associated with inflammatory signaling pathways, including NF-κB, in patients with asthma and COPD [11]. While our study focused on the phenotypic changes and cytokine levels, the observed reduction in these markers by GOG suggests a potential modulation of upstream inflammatory pathways, which warrants further direct mechanistic investigation. GOG decreased TNF-α and IL-6 expression, suggesting its therapeutic potential in the COPD pathology model. We then conducted in vivo experiments using a mouse model with CSE- and LPS-induced lung damage. We analyzed the number of neutrophils using cytospin in BALF isolated from COPD mice. The number of neutrophils significantly increased in the control group, whereas it significantly decreased in the GOG treatment groups. This result is consistent with previous studies demonstrating an increase in the number of neutrophils in the BALF isolated from the control group [21,22]. An increased number of active neutrophils in smokers and patients with COPD is strongly associated with accelerated lung function decline due to airway blockage [21]. Neutrophils also play an important role in airway inflammation in the acute exacerbation of COPD [47]. Collectively, these results indicate that GOG may play a role in suppressing the accumulation of neutrophils, which are key contributors to COPD pathology.

In the present study, ELISA analysis of BALF collected from the COPD-induced mouse model revealed that TNF-α and IL-17A expression levels were significantly increased in the control group compared to those in the normal group, whereas they were significantly decreased in the GOG treatment groups compared to those in the control group. The sputum of patients with COPD [48] and the serum of those with COPD-associated cachexia [49] exhibit elevated TNF-α levels. Notably, IL-17A prolongs the survival of neutrophils [50] and is involved in their accumulation in the peripheral airway of long-term smokers [51]. These results suggest that GOG inhibits neutrophil-mediated inflammation by regulating the expression of related cytokines. In addition, treatment with GOG suppressed the increased expression of CXCL-1 and MIP2, which are mainly secreted by macrophages and serve as chemokines, inducing the accumulation of inflammatory and immune cells, including neutrophils, at a specific area [52]. These results suggest that GOG may potentially suppress the infiltration of inflammatory cells into lung tissue by reducing the expression levels of CXCL-1 and MIP2. Moreover, RT-PCR analysis of lung tissue isolated from COPD mice revealed significantly increased *Mip2*, *Cox-2*, and *Trpv1* mRNA expression in the control group compared to the normal group. However, their expression levels were significantly decreased in the GOG treatment group compared to those in the control group. COX-2 is a major inflammation mediator, while TrpV1 is involved in the COPD-induced inflammatory response by increasing the expression of genes related to oxidative stress and inflammation [41]. Therefore, these results suggest that by inhibiting such processes, GOG may regulate the inflammatory response.

Interestingly, in several parameters such as Figure 5B and Figure 7D,F, GOG at a dose of 200 mg/kg exhibited higher efficacy than at 400 mg/kg. Interestingly, in several parameters (e.g., Figure 5B and Figure 7D,F), GOG at 200 mg/kg exhibited higher efficacy than at 400 mg/kg. While both doses significantly improved the pathological markers compared to the control group, the observed non-linear response suggests that the therapeutic window of GOG in this model may be optimal at 200 mg/kg. Such non-monotonic dose-responses are documented in complex herbal formulations, where multiple bioactive compounds may exert synergistic or antagonistic interactions depending on the concentration. Although we cannot definitively confirm the underlying mechanism without further pharmacokinetic or binding affinity data, this phenomenon might be related to the saturation of specific signaling pathways or a complex interplay between the diverse components of GOG. Future studies employing a broader range of doses and mechanistic modeling are required to clarify this observation. These findings indicate that 200 mg/kg may represent the optimal therapeutic dose for mitigating lung injury in this experimental setting, and further studies are needed to elucidate the precise molecular thresholds of GOG’s multi-component interactions. Mouse Siglec-F is an eosinophil surface receptor [53]. CD11b^+^/Gr-1^+^ is a granulocyte-specific cell surface protein, which induces the secretion of allergy mediators, such as histamine, involved in the production of cytokines, including IL-4, IL-5, and IL-13, thereby aggravating inflammation [54]. The activation of CD4^+^ T cells, which play a major role in COPD pathology, leads to a CD44^high^ and CD62L^−^ phenotype. CD44 is essential for the migration of lymphocytes to the inflammation site [55], and neutrophils induce a phenotype caused by downregulated CD62L expression in COPD [56]. CD21, CD35, and B220 are expressed at a specific stage of B cell differentiation and activation and are thus used as indicators for B cell activation [57]. GOG treatment inhibited immune cell expression in BALF and lung tissues, suggesting its effects on pathological immune response mediated by immune cells in COPD.

In the present study, histological analysis of lung tissue extracted from the COPD mouse model revealed relatively consistent and small alveoli in the normal group. However, extended alveoli were frequently observed in the control group, exhibiting inconsistent morphology, along with collagen deposition, airway wall hyperplasia, and infiltration of a large number of inflammatory cells around the alveoli. Such changes reflect significant alveolar enlargement and airway remodeling, which are characteristic pathological features observed in COPD-like lung injury [7]. While the 21-day model provides insight into the inflammatory and structural responses following CSE and LPS insult, we acknowledge that without a prolonged recovery period, these observations should be interpreted as representative of acute-to-subacute structural damage rather than confirmed irreversible remodeling. Nevertheless, GOG treatment effectively mitigated these morphological alterations, suggesting its protective potential against lung tissue destruction. However, inflammatory cell infiltration was reduced, and the size and morphology of the alveoli were consistent in the positive control group and GOG treatment groups. In addition, quantitative analysis revealed that GOG significantly alleviates tissue damage, suggesting its protective role against lung injury. Overall, GOG inhibited the activation of inflammatory cytokines and immune cells in the COPD mouse model and protected the lung by mitigating lung tissue damage, suggesting its therapeutic effects on COPD.

In conclusion, we assessed the effects of GOG on COPD, analyzed the cytokines and immune cells associated with lung damage, and investigated histological changes in a CSE- and LPS-induced COPD mouse model. We found that GOG significantly suppressed neutrophil accumulation and TNF-α, IL-17A, MIP2, and CXCL-1 expression in the BALF of mice. It also significantly decreased the mRNA expression of *Mip2*, *Cox-2,* and *Trpv1* in lung tissue. Treatment with GOG significantly decreased the number of macrophages and Gr-1^+^/Siglec-F, Gr-1^+^/CD11b^+^, and CD44^high^/CD62L^−^ cells in the BALF. It also significantly decreased the number of neutrophils and Gr-1^+^/Siglec-F, Gr-1^+^/CD11b^+^, CD44^high^/CD62L^−^, and CD21^+^/CD35^+^/B220^+^ cells and the histological analysis scores for lung damage. Overall, our findings suggest that GOG suppresses lung damage by regulating immune cells and inflammatory cytokines in COPD mice.

## 4. Materials and Methods

The Gyeongok-go (GOG) used in this study was standardized and provided by the Daejeon Oriental Medicine Hospital of Daejeon University (Daejeon, Republic of Korea). The GOG was prepared by mixing the herbal ingredients (Table 1). To ensure consistency in experimental conditions, a single batch of GOG was used throughout the study. For both in vitro and in vivo administration, GOG was dissolved in sterile distilled water or phosphate-buffered saline (PBS). To ensure sterility for cell culture experiments, the GOG solution was filtered through a 0.22-μm syringe filter (Millipore, Billerica, MA, USA) to remove any microbial contamination. The concentration of the extract was calculated based on the dry weight of the final formulation. The GOG used in this study was prepared according to the classical ratio described in the Donguibogam, a representative traditional Korean medical text. This fixed ratio was selected to ensure the standardization and reproducibility of the experimental results, as it represents the most widely utilized formulation in clinical practice for respiratory diseases. The specific combination is designed to exert synergistic anti-inflammatory and lung-protective effects, which are critical for addressing the complex pathology of COPD.

### 4.1. Reagents and Instruments

LPS was purchased from Sigma-Aldrich (St. Louis, MO, USA). Mouse IL-6, mouse TNF-α, mouse MIP2, mouse CXCL-1, and mouse IL-17 were purchased from R&D Systems (Minneapolis, MN, USA). Fetal bovine serum (FBS) was obtained from Serum Source International Inc. (Charlotte, NC, USA). Formaldehyde, RPMI1640 medium, and Dulbecco’s phosphate-buffered saline (D-PBS) were purchased from Sigma-Aldrich. All other reagents used were of special grade.

### 4.2. UPLC with QTOF-MS Analysis

For major compound analysis, 1.0 g of crushed GOG was extracted into 20 mL of a methanol/water mixture (4:1, *v*/*v*) using a sonicator (Jeiotech, Seoul, Republic of Korea) for 30 min and centrifuged at 3000 rpm for 5 min (Eppendorf, Hamburg, Germany). Exactly 1 mL of supernatant was filtered through a 0.2-μm PTFE filter and subjected to UPLC-QTOF-MS analysis (Waters Corp., Milford, MA, USA). Aliquots (3.0 μL) of each sample were then injected into a BEH C_18_ column (2.1 × 100 mm, 1.7 μm) at a flow rate of 0.4 mL/min. The two mobile phases were prepared as follows: A, water with 0.1% formic acid; B, acetonitrile with 0.1% formic acid. Assay conditions were as follows: 0–1 min, 10% B; 1–17 min, 10–40% B; 17–21 min, 85% B; 21–21.3 min, 100% B; 21.3–23.3 min, 100% B; 23.3–23.4 min, 100–10% B; 23.4–25 min, 10% B, and back to 10% B. Mass spectrometry (Vion IMS QTof, Waters Corp.), equipped with an electrospray ionization (ESI) interface, was performed in negative ion mode ([M−H]^−^). Nitrogen (N_2_) was used as the desolvation gas, and the desolvation temperature was set to 350 °C at 600 L/h with a source temperature of 110 °C. The capillary and cone voltages were set to 2500 and 40 V, respectively. A sprayer with a reference solution of leucine-enkephalin ([M−H]^−^ *m*/*z* 554.2615) was used as the lock mass. All extraction and chromatographic solvents were of LC-MS grade (Merck Millipore, Burlington, MA, USA).

### 4.3. Cell Culture

MH-S cells were purchased from the Korean Cell Line Bank (Seoul, Republic of Korea). For cell culture, RPMI1640 medium with L-glutamine containing 1% penicillin–streptomycin and 10% FBS was used. The cells were incubated at 37 °C with 5% CO_2_.

### 4.4. Measurement of Inflammatory Cytokines

The effects of GOG on the expression of TNF-α and IL-6 in MH-S cells treated with LPS were measured using ELISA. Different concentrations of GOG extract at 50, 100, 200, and 400 µg/mL were added to the wells (2 wells for each concentration) for 1 h, followed by incubation with 100 ng/mL of LPS for 24 h. Subsequently, capture antibodies were mixed into the coating buffer, and 100 µL of this mixture was added to each well and incubated overnight at 4 °C. After washing four times with washing buffer, 200 µL of the assay diluent was added to each well, blocked for 1 h at room temperature, and washed four times with washing buffer. After 2–10-fold dilution with assay diluent, 100 µL of each capture antibody was added to the coated 96-well plate and incubated for 2 h at room temperature. After washing four times with washing buffer, 100 µL of biotin-conjugated antibody was added to each well and allowed to react for 1 h at room temperature. After washing four times, 100 µL streptavidin–horseradish peroxidase (HRP) solution was added for 1 h at room temperature, followed by four washes with washing buffer. Subsequently, 100 µL of the substrate solution was added for 5–30 min, and 50 µL of the stop solution was finally added to terminate the reaction. Absorbance was then measured at 450 nm.

### 4.5. Animal Experiments

C57BL/6 mice (6–8 weeks old) were obtained from Orient Bio Inc. (Seongnam, Republic of Korea) and used after 1 week of acclimatization. The mice were housed under pathogen-free conditions with a standard laboratory diet and maintained at a temperature of 22–24 °C, a humidity of 50 ± 10%, and controlled day and night cycles. To minimize selection bias, mice were randomly assigned to each experimental group using a simple randomization method. Furthermore, all outcome assessments—including the histological scoring of lung tissues and the differential cell counting in BALF—were performed by investigators who were blinded to the treatment groups. The group assignments were only revealed after the completion of all data analyses to ensure the objectivity and integrity of the results. The animal protocol was approved by the committee for animal welfare at Daejeon University (DJUARB2021-024). Data are expressed as the mean ± SEM of at least two independent experiments performed in triplicate. For animal studies, 8 mice per group were utilized to ensure statistical relevance. All experimental procedures were performed following the Guide for the Care and Use of Laboratory Animals of the National Institute of Health and guidelines of the Institutional Animal Care and Use Committee of the South Korea Research Institute of Bioscience and Biotechnology (Daejeon, Republic of Korea). The animal study protocol was reviewed and approved by the Institutional Animal Care and Use Committee (IACUC) of Daejeon University (Protocol Code: DJUARB2021-024, Date of Approval: 24 December 2021). All experiments were performed in accordance with the relevant guidelines and regulations for animal welfare.

### 4.6. Preparation of the CSE

CM7 (CORESTA-approved Monitor No. 7) reference cigarettes were conditioned at 22 ± 2 °C and relative humidity of 60 ± 5% according to ISO conditions (one puff/min, 35 mL puff volume over 2 s, every 60 s) for 48 h or more. The cigarettes were smoked using an automatic smoking machine (Borgwaldt RM20; Heinr. Borgwaldt, Germany) following ISO conditions (puff volume, 35 mL; duration, 60 s; interval, 2 s). Cigarette smoke was trapped on a Cambridge filter pad (0.22 μm, Ø4 mm) and extracted with isopropanol. Total particulate matter (TPM) was prepared for the entire 2-year period and stored at −80 °C. TPM was tested immediately after preparation (T0), after 1 month (T1), and after 3 months (T3). TPM content (mg/cig) in standard tobacco mainstream smoke was calculated using the following equation:

(1) $$TPM=\frac{W_{FHA}-W_{FHB}}{N}$$

where *W_FHA_* is the weight of the filter holder after smoking, *W_FHB_* is the weight of the filter holder before smoking, and *N* is the number of cigarettes smoked per trap (cig.).

### 4.7. Establishment of the COPD Mouse Model

**Figure 10:**
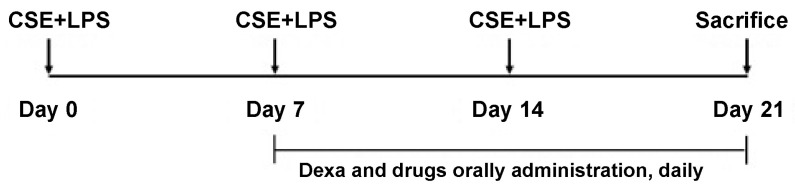
Effects of GOG on the histology of the lungs of mice with COPD. Mice were induced via CSE+LPS aspiration (Control) and treated with dexamethasone 3 mg/kg (Dexa) and GOG (100, 200, 400 mg/kg) for 21 days.

A COPD mouse model was established via exposure to CSE and LPS (Sigma-Aldrich), as depicted in Figure 10. Following intraperitoneal injection of anesthetics, CSE (1 mg/mL) and LPS (100 µg/mL) were administered via intratracheal injection 3 times at 7 d intervals. Dexamethasone and all other drugs were orally administered daily for 14 d. The mice were divided into the following groups: (1) C57BL/6_Nr (no treatment), (2) control group (CSE/LPS), (3) dexamethasone group (positive control, CSE/LPS + Dexa 3 mg/kg), (4) GOG-100 group (CSE/LPS + GOG 100 mg/kg), (5) GOG-200 group (CSE/LPS + GOG 200 mg/kg), and (6) GOG-400 group (CSE/LPS + GOG 400 mg/kg).

### 4.8. Isolation of the BALF

After the completion of animal experiments, the mice were euthanized, and their chest was opened to expose the airway. A catheter was inserted into the trachea and ligated and fixated with a string. Subsequently, 1 mL of Dulbecco’s modified Eagle medium (DMEM) without FBS was added to the lung and circulated three times before isolating the BALF. BALF was centrifuged for 5 min at 1750 rpm and 4 °C, and the supernatant was refrigerated separately for cytokine analysis. The cells isolated from the BALF were treated with an ammonium–chloride–potassium solution for 3 min to dissolve the red blood cells and washed with DMEM without FBS. The total number of cells was then counted using a hemocytometer.

### 4.9. Measurement of the Total Number of Neutrophils in the BALF

The total number of neutrophils in the BALF was measured using cytospin. Precipitated blood cells were isolated, and Diff-Quick staining was performed three times. The neutrophils were quantified under a 400× optical microscope (Nikon, Tokyo, Japan).

### 4.10. ELISA

The concentrations of IL-17A, TNF-α, MIP2, and CXCL-1 in the bronchoalveolar lavage fluid (BALF) were quantified using commercially available DuoSet^®^ ELISA kits (R&D Systems, Minneapolis, MN, USA) according to the manufacturer’s instructions. Briefly, 96-well microplates were coated with the respective capture antibodies overnight at room temperature. After blocking with 1% BSA in PBS for 1 h, standard dilutions and BALF samples were added to the wells and incubated for 2 h. Following a series of washes to remove unbound proteins, biotinylated detection antibodies were added. The signals were amplified by the addition of streptavidin-conjugated horseradish peroxidase (HRP). The colorimetric reaction was developed using a substrate solution (TMB) and terminated by adding a stop solution H_2_SO_4_. The optical density (OD) of each well was measured at 450 nm using a microplate reader. The concentration of each cytokine was calculated by interpolation from a standard curve generated using recombinant proteins provided in the kits. All samples were analyzed in duplicate, and the results were expressed in pg/mL.

### 4.11. RT-PCR

RT-PCR was performed to measure the mRNA expression levels of *Mip2*, *Cox2*, and *Trpv1* in lung tissues. The lung tissues were extracted from mice and pulverized in 500 mL of RNAzol (CS-105B; Tel-Test, Inc., Friendswood, TX, USA) until dissolution. RT-PCR of the synthesized cDNAs was performed using the Applied Biosystems 7500 Fast Real-Time PCR system (Applied Biosystems, Foster City, CA, USA) and Power SYBR Green PCR Master Mix (Applied Biosystems). Mouse glyceraldehyde-3-phosphate dehydrogenase (*G3pdh*) cDNA probe (Applied Biosystems) was used as a control. The primer and sequences for the probes are presented in Table 2. *G3pdh* was used as an internal reference. Relative quantitative expression was calculated by calculating y = x(1 + e)n of the target group, where y is the yield, x is the starting quantity, e is the efficiency, and n is the number of cycles.

### 4.12. Flow Cytometry of Immune Cells in BALF

To analyze immune cell populations, BALF cells or lung tissues were suspended in staining buffer (PBS containing 1% BSA). Cells were stained with the following fluorochrome-conjugated antibodies: Gr-1-PE, Siglec-F-FITC, CD11b-APC, CD44-FITC, and CD62L-PE (all from BD Biosciences, San Jose, CA, USA or eBioscience, San Diego, CA, USA). After incubation for 30 min at 4 °C in the dark, cells were washed and analyzed using a Flow Cytometer (BD FACSCanto II, BD Biosciences). Data were processed using FlowJo software (version 10, Tree Star, Inc., Ashland, OR, USA).

### 4.13. Hematoxylin and Eosin and Masson’s Trichrome Staining

To assess the level of lung damage and examine the bronchiole and alveolar inflammation and infiltration of blood cells, hematoxylin and eosin and Masson’s trichrome staining were performed. For hematoxylin and eosin staining, the slides were attached to the cover slides and observed under a 200× optical microscope (Nikon). To quantitatively assess the severity of lung tissue damage, a histological activity index was employed based on previously established methods with minor modifications. The lung injury was evaluated across six distinct pathological parameters: (1) inflammatory cell infiltration, (2) blood vessel congestion and hemorrhage, (3) bronchiolar wall thickening, (4) alveolar destruction and emphysematous changes, (5) collagen deposition, and (6) goblet cell hyperplasia. Each parameter was graded on a scale of 0 to 2 according to the degree of damage: 0 for normal, 1 for mild to moderate damage, and 2 for severe damage. The total lung injury score was calculated by summing the scores of all six categories, with a maximum possible score of 12. Histological sections were independently evaluated by a researcher blinded to the experimental groups to ensure objectivity.

### 4.14. Immunohistochemistry

Lung tissues were fixed in 4% paraformaldehyde, embedded in paraffin, and sectioned at 4 μm. After deparaffinization and rehydration, antigen retrieval was performed by heating in 10 mM citrate buffer (pH 6.0). Sections were blocked with 5% normal goat serum for 1 h to prevent non-specific binding. The slides were incubated overnight at 4 °C with a primary antibody against rabbit anti-Filaggrin (ab24584, 1:200; Abcam, Cambridge, UK). After washing, sections were incubated with a FITC-conjugated secondary anti-rabbit IgG antibody (1:500; Abcam) for 1 h at room temperature. Nuclei were counterstained with DAPI. Images were captured using a fluorescence microscope (Zeiss LSM 510; Carl Zeiss, Oberkochen, Germany).

### 4.15. Statistical Analysis

Data are expressed as the mean ± standard error of the mean (SEM). Statistical differences between two groups were analyzed using the unpaired Student’s t-test, while comparisons among multiple groups were performed using one-way analysis of variance (ANOVA) followed by Dunnett’s post hoc test for multiple comparisons. All statistical analyses were conducted using SPSS software (version 19.0; IBM, USA), and a *p*-value < 0.05 was considered statistically significant.

## 5. Conclusions

In summary, this study demonstrates that Gyeongok-go (GOG) exerts significant protective effects against lung injury in an LPS- and cigarette smoke-induced COPD mouse model. GOG effectively suppressed the inflammatory response in macrophages and reduced the infiltration of inflammatory cells, such as neutrophils, into the lung tissues and bronchoalveolar lavage fluid. By downregulating the expression of pro-inflammatory cytokines (TNF-α, IL-6, IL-17A) and key inflammatory genes (Mip2, Cox-2, Trpv1), GOG alleviated the severity of pathological lung damage. These findings suggest that GOG holds strong potential as a natural therapeutic agent for managing and preventing the progression of COPD, providing a scientific basis for its clinical application in respiratory health.

## Figures and Tables

**Table 1:** Composition of Gyeongok-go (GOG).

Herb	Pharmacognostic Name	Amount (g)
Saengjihwang	*Rehmanniae Radix Recens*	7.27
Insam	Ginseng Radix	0.68
Bokryeong	*Poria Sclerotium*	1.36
Bongmil	*Mel*	4.54
Total amount		13.86

**Table 2:** Oligonucleotide sequences used for mouse real-time polymerase chain reaction.

Gene	Primer	Sequence
*Mip2*	Forward	5′-TCCAGAGCTTGAGTGTGACG-3′
	Reverse	5′-GCCCTTGAGAGTGGCTATGA-3′
*Cox-2*	Forward	5′-TTCAAATGAGATTGTGGGAAAAT-3′
	Reverse	5′-AGATCATCTCTGCCTGAGTATCTT-3′
*Trpv1*	Forward	5′-GGCTGTCTTCATCATCCTGCTGCT-3′
	Reverse	5′-GTTCTTGCTCTCCTGTGCGATCTTGT-3′
*G3pdh*	VIC	5′-TGCATCCTGCACCACCAACTGCTTAG-3′

## Data Availability

The original contributions presented in this study are included in the article. Further inquiries can be directed to the corresponding author.

## Supplementary Materials

The following supporting information can be downloaded at https://www.mdpi.com/article/10.3390/ph19040618/s1, Table S1: Characterization and tentative identification of major compounds identified in Gyeongok-go extract using ultra-performance chromatography with quadrupole time-of-flight mass spectrometry analysis.

## Abbreviations

The following abbreviations are used in this manuscript:

GOG	Gyeongok-go
COPD	Chronic obstructive pulmonary disease
BALF	Bronchoalveolar lavage fluid
TNF-α	Tumor necrosis factor-alpha
IL-	Interleukin
ELISA	Enzyme-linked immunosorbent assay
LPS	Lipopolysaccharide
CXCL-1	Chemokine ligand-1
CSE	Cigarette smoke extract
RT-PCR	Real-time polymerase chain reaction
