# Supplementary material for: Inhibitory Effects of Gyeongok-go on Lung Injury in a Chronic Obstructive Pulmonary Disease Mouse Model
Source: Pharmaceuticals (Basel). 2026 Apr 14;19(4):618. doi: 10.3390/ph19040618 (PMC13119437; doi:10.3390/ph19040618)
Supplement: Supplementary file 1 [file pharmaceuticals-19-00618-s001.zip › pharmaceuticals-4163746-supplementary.pdf]

Table S1. Characterization and tentative identification of major compounds identified in Gyeongok-go extract using ultra-performance chromatograph with quadrupole time-of-flight mass spectrometry analysis.

Peak No.[a]	ESI-MS R_T (min)	Detected ion [M-H]- (*m/z*)	Detected ion [M-H+HCOOH]- (*m/z*)	Error (ppm)	Molecular formula	Fragments
1	11.9	637.4514	683.4390	2.9	$C_{36}H_{62}O_9$	475
2	12.1	783.5143	829.4955	0.7	$C_{42}H_{72}O_{13}$	637, 475
3	12.3	637.4674	683.4380	1.5	$C_{36}H_{62}O_9$	475
4	12.4	955.4926	–	2.4	$C_{48}H_{76}O_{19}$	793
5	15.4	783.5143	829.4976	3.3	$C_{42}H_{72}O_{13}$	621
6	16.2	765.5036	811.4868	3	$C_{42}H_{70}O_{12}$	619, 457
7	16.3	619.4407	665.4282	2.6	$C_{36}H_{60}O_8$	–
8	16.7	619.4407	665.4274	1.4	$C_{36}H_{60}O_8$	–
9	16.9	793.4387	–	1.6	$C_{42}H_{66}O_{14}$	–
10	17.5	783.5178	829.4965	1.9	$C_{42}H_{72}O_{13}$	621, 459
11	17.7	783.5178	829.4960	1.3	$C_{42}H_{72}O_{13}$	621, 459
12	18.8	471.3476	–	0.4	$C_{30}H_{48}O_4$	–
13	19.1	483.3112	–	0.4	$C_{30}H_{44}O_5$	409, 397
14	19.2	483.3474	–	0	$C_{31}H_{48}O_4$	437, 325
15	19.3	765.5071	811.4853	1.1	$C_{42}H_{70}O_{12}$	603
16	19.4	765.5071	811.4850	0.7	$C_{42}H_{70}O_{12}$	603
17	19.6	497.3267	–	0	$C_{31}H_{46}O_5$	423
18	19.7	485.3268	–	0.2	$C_{30}H_{46}O_5$	469, 397
19	20.0	481.3315	–	-0.6	$C_{31}H_{46}O_4$	–
20	20.3	483.3481	–	1.4	$C_{31}H_{48}O_4$	–
21	21.2	513.3587	–	1.4	$C_{32}H_{50}O_5$	–
22	21.4	525.3587	–	1.3	$C_{33}H_{50}O_5$	–
23	21.6	527.3740	–	0.8	$C_{33}H_{52}O_5$	–
24	22.4	453.3371	–	0.4	$C_{30}H_{46}O_3$	–

[a], the peak no. corresponds to the peak no. in Figure 2.
